# The current and future global distribution and population at risk of dengue

**DOI:** 10.1038/s41564-019-0476-8

**Published:** 2019-06-10

**Authors:** Jane P. Messina, Oliver J. Brady, Nick Golding, Moritz U. G. Kraemer, G. R. William Wint, Sarah E. Ray, David M. Pigott, Freya M. Shearer, Kimberly Johnson, Lucas Earl, Laurie B. Marczak, Shreya Shirude, Nicole Davis Weaver, Marius Gilbert, Raman Velayudhan, Peter Jones, Thomas Jaenisch, Thomas W. Scott, Robert C. Reiner, Simon I. Hay

**Affiliations:** 1 School of Geography and the Environment, University of Oxford, Oxford, UK; 2 School of Interdisciplinary Area Studies, University of Oxford, Oxford, UK; 3 Centre for the Mathematical Modelling of Infectious Diseases, London School of Hygiene & Tropical Medicine, London, UK; 4 Department of Infectious Disease Epidemiology, London School of Hygiene & Tropical Medicine, London, UK; 5 School of BioSciences, University of Melbourne, Parkville, VIC Australia; 6 Harvard Medical School, Harvard University, Boston, MA USA; 7 Boston Children’s Hospital, Boston, MA USA; 8 Department of Zoology, University of Oxford, Oxford, UK; 9 Environmental Research Group Oxford, c/o Department of Zoology, University of Oxford, Oxford, UK; 10 Institute for Health Metrics and Evaluation, University of Washington, Seattle, WA USA; 11 Big Data Institute, Li Ka Shing Centre for Health Information and Discovery, University of Oxford, Oxford, UK; 12 Université Libre de Bruxelles, Brussels, Belgium; 13 World Health Organization, Geneva, Switzerland; 14 Waen Associates Ltd, Y Waen, Islaw’r Dref, Dolgellau, Gwynedd, UK; 15 Department of Infectious Diseases, Section Clinical Tropical Medicine, Heidelberg University Hospital, Heidelberg, Germany; 16 Department of Entomology and Nematology, University of California, Davis, USA

**Keywords:** Infectious diseases, Diseases

## Abstract

Dengue is a mosquito-borne viral infection that has spread throughout the tropical world over the past 60 years and now affects over half the world’s population. The geographical range of dengue is expected to further expand due to ongoing global phenomena including climate change and urbanization. We applied statistical mapping techniques to the most extensive database of case locations to date to predict global environmental suitability for the virus as of 2015. We then made use of climate, population and socioeconomic projections for the years 2020, 2050 and 2080 to project future changes in virus suitability and human population at risk. This study is the first to consider the spread of *Aedes* mosquito vectors to project dengue suitability. Our projections provide a key missing piece of evidence for the changing global threat of vector-borne disease and will help decision-makers worldwide to better prepare for and respond to future changes in dengue risk.

## Abstract (web summary)

Statistical mapping techniques provide insights into the current geographical spread of the mosquito-borne dengue virus infection and predict changes in the areas that will be environmentally suitable to the virus for the years 2020, 2050 and 2080.

## Main

Dengue causes the greatest human disease burden of any arbovirus, with an estimated 10,000 deaths^1^ and 100million symptomatic infections per year^2^ in over 125 countries^1^. Roughly half of the global population currently lives in areas that are environmentally suitable for dengue transmission^3,4^. Dengue is transmitted to humans by *Aedes* species mosquitoes, which thrive in tropical and sub-tropical urban centres around the globe^5^. In combination with these global trends, rising temperatures attributed to climate change have increased concerns that dengue will intensify in already endemic areas through faster viral amplification, increased vector survival, reproduction and biting rate, ultimately leading to longer transmission seasons and a greater number of human infections, more of which are expected to be severe^6,7^. Increasing temperatures may further exacerbate this situation by enabling greater spread and transmission in low-risk or currently dengue-free parts of Asia, Europe, North America and Australia^8,9^.

To anticipate such trends, make investment cases for alternative modes of sustained vector control, and plan mitigation strategies, it is essential that public health policymakers, vaccine developers and vector control specialists be provided with robust estimates of the current and future global distribution of dengue. While all efforts to produce such estimates have so far projected an increase in the overall global extent of dengue transmission, there is a lack of agreement regarding specific geographic patterns of expansion and an absence of insight on the potential for contraction^5^. Uncertainty intervals have not commonly been provided for estimates, making it more difficult to reconcile causes of differences and to provide context for policymakers. Only one previous study incorporated non-meteorological variables^10^ into its model, and none have validated their models using the now extensively cited global distributions of the *Aedes* mosquito vector^5^. Historically, it is the spread of *Aedes* mosquitoes that has driven expansion of dengue. Most existing global dengue projections anticipate widespread transmission in Europe inconsistent with the current restricted distribution of *Aedes aegypti* on the continent^1^. This is a crucial concern for estimating the future population at risk and public health priority of dengue globally.

Building on previous methods successfully applied to map a 2010 global distribution of dengue^3^, we implemented species distribution modelling to produce a 2015 map of environmental suitability (see Methods). Our map incorporates an update of more recent documented occurrences and new socioenvironmental covariates arising from improvements in data quality and availability. We also present projections of the global distribution of dengue in 2020, 2050 and 2080, which address previous limitations by using (1) the most exhaustive and spatially detailed compendium of dengue occurrence locations to date to validate the fitted models; (2) a comprehensive set of socioeconomic and environmental covariates with projections based on the newest Relative Concentration Pathway (RCP) scenarios and Shared Socioeconomic Pathways (SSPs) from the Intergovernmental Panel on Climate Change (IPCC); and (3) an ensemble species distribution modelling procedure. Most importantly, our maps account for the present and future distributions of *Aedes* mosquito vectors, which have not previously been incorporated into projections for dengue.

**Fig. 1:**
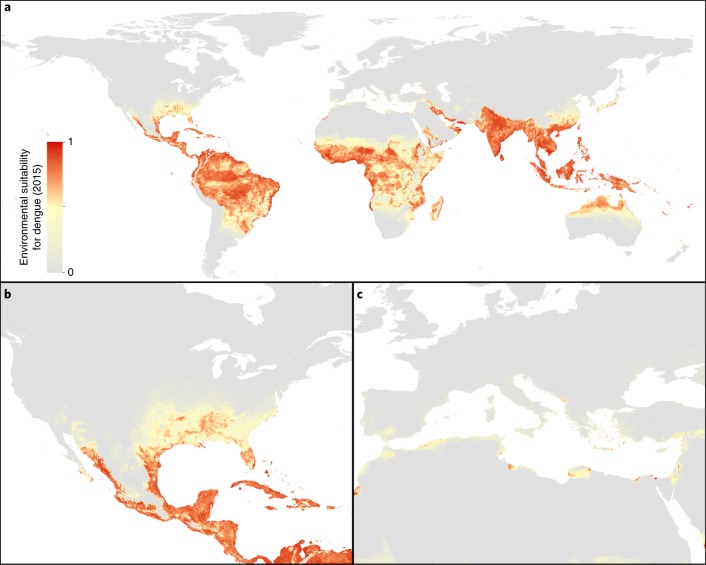
Probability of occurrence of dengue in 2015. **a**–**c**, Maps displaying global probability data (**a**) as well as data for North America and parts of Central America and the Caribbean (**b**) and North Africa, the Middle East and Europe (**c**). Values range from 0 (grey, unsuitable environment) to 1 (red, suitable environment).

**Fig. 2:**
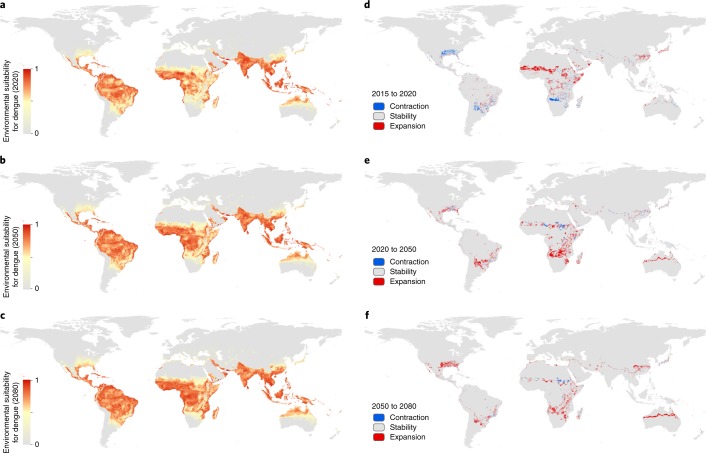
Environmental suitability for dengue occurrence according to RCP6.0 and SSP2. **a**–**c**, Projected data shown for 2020 (**a**), 2050 (**b**) and 2080 (**c**). **d**–**f**, Changes in areas classified as at-risk (using the suitability threshold of 0.467).

Specifically, we fit a boosted regression tree (BRT) statistical model based on a total of 13,604 dengue occurrence locations between the years 1960 and 2015 and the following set of covariates to accurately characterize the distribution of dengue: (1) temperature suitability for dengue transmission^11^; (2) cumulative annual precipitation; (3) minimum relative humidity; (4) gross domestic product (GDP) per capita; (5) human population density; (6) environmental suitability for *Aedes aegypti* from ref. ^12^; and (7) environmental suitability for *Aedes albopictus*^12^. The result is a 5 × 5-km resolution global map of environmental suitability (an index ranging from 0 to 1) for dengue occurrence. Environmental suitability is defined as the conditional probability of observing dengue over a long-term average given the state of the environment at a location^13^. The BRT model was iterated 100 times to allow for estimation of uncertainty in individual BRT predictions. The mean of this ensemble of BRTs was then mapped (Fig. 1). We then made 100 projections for the years 2020, 2050 and 2080 for each of three climate scenarios defined by the IPCC and three related socioeconomic scenarios, producing a total of nine final mean projections (with 95% credible intervals) of the global distribution of dengue that can be assessed in the context of progress towards the emission scenario commitments made during the 2015 Paris climate accord^14^. The projected maps for RCP6.0 and SSP2 (see Methods), which assume a relatively moderate increase in CO_2_, are shown in Fig. 2a–c.

An overview of the modelling and projection procedures can be found in Supplementary Fig. 1, with the projected maps for RCP4.5/SSP1 and RCP8.5/SSP3 for all years available via Figshare. Supplementary Fig. 2a shows the locations of the 13,604 standardized occurrence records in our final dataset, accompanied by the graph in Supplementary Fig. 2b, which shows the number of reported occurrence locations globally by year and region. We restricted our models to make predictions only within areas where we predict the occurrence and future potential spread of *Ae. aegypti* or *Ae. albopictus* populations^12^. We also restricted predictions to exclude areas where extreme high or low temperatures prevent the mosquito from surviving long enough to allow autochthonous dengue virus (DENV) transmission at any time of the year^11,15^.

Our models continue to predict high levels of environmental suitability for DENV in many areas within the tropical and sub-tropical zones in 2015, with a distribution that is largely consistent with currently known areas of endemicity in South America, Southeast Asia and central Africa^3,4,16^ (Fig. 1). We predict that 3.83 (3.45–4.09) billion people (roughly 53% of the global population) live in areas that are suitable for dengue transmission, with the vast majority in Asia, followed by Africa and the Americas. Our models showed DENV suitability to be particularly influenced by temperature suitability for transmission, annual cumulative precipitation, relative humidity and GDP, contributing 67.8%, 13.4%, 5.8% and 5.3% to the variation in the ensemble of models, respectively. Partial dependence plots showing the average effect of each covariate on the overall response are shown in Supplementary Fig. 3. Validation statistics indicated high predictive performance of the BRT ensemble mean map evaluated using a more stringent 10-fold cross-validation spatial sorting bias corrected procedure (see Methods), with an area under the receiver operating characteristic curve (AUC) of 0.72 (credible interval (CI) 0.69–0.75, equivalent to an AUC of 0.95 without pairwise distance sampling).

**Fig. 3:**
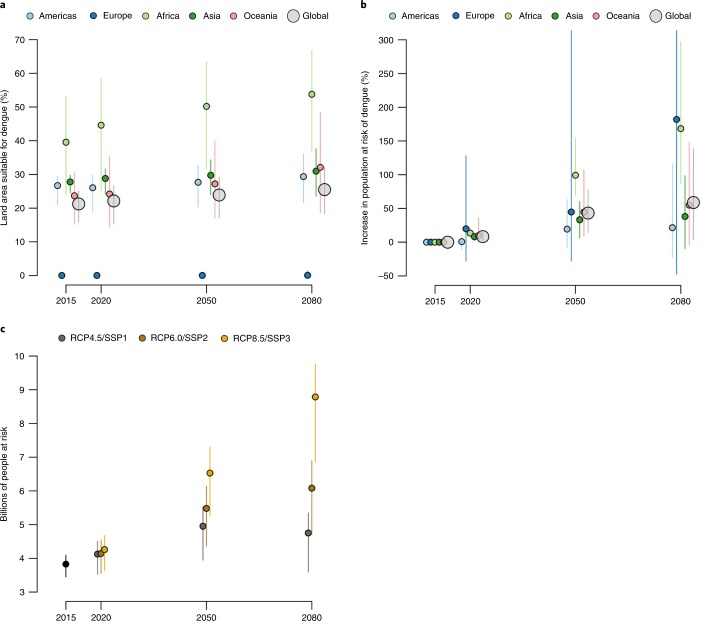
Projected changes in the risk of dengue occurrence. **a**–**c**, There are predicted changes in the land area (**a**) and population (**b**,**c**) at risk of dengue in the future. Mean and 95% credible intervals are shown for each ensemble of 100 BRT models.

Yearly climate variability means that some years are more unsuitable for dengue transmission in some areas than others, and this will continue to be the case through to 2080. As such, the projections we provide are meant to show average trends and should not be interpreted as predictive for specific years. Overall, we predict minimal changes in the total global area at risk of dengue, but significant subnational changes in risk distributions between 2015 and 2080 (Fig. 2). Much of the southeastern USA is predicted to become suitable by 2050, and dengue risk is predicted to extend to higher altitudes in central Mexico, northern areas of Argentina and inland areas of Australia (Fig. 1). Many of the larger cities in coastal eastern China and Japan are also likely to become suitable by 2050. The continent that is likely to see the biggest change in dengue risk is Africa, where large increases in suitability are predicted in southern Africa, and into the Sahel in West Africa, largely due to more favourable temperatures and increased rainfall (Figs. 2 and 3a,b). The Sahel currently only sporadically reports dengue^4^, and many areas within this region are likely to remain the least capable to detect, respond to and control the risk of dengue outbreaks^17^. At the same time, there are a number of areas where we predict contraction of dengue suitability. Some areas in central East Africa are predicted to see declining suitability as they become hotter and drier, and India is also projected to have a slight decline in dengue suitability although will remain at high levels of suitability. These settings illustrate the constraints imposed by the upper thermal limits of dengue, where consistently high temperatures (over 35 °C; Supplementary Fig. 6) see rapid declines in DENV transmission, principally due to reduced mosquito survival^11,18^.

In contrast to other studies, we do not predict significant spread of dengue risk across continental Europe over the coming decades. Despite increasingly suitable temperatures for vectors and DENV, projected reductions in precipitation and relative humidity, and the absence of the more competent *Ae. aegypti* vector in the Mediterranean region, are likely to lead to only very modest increases in dengue suitability. Some isolated coastal areas around Turkey and southern Spain may see dengue risk grow to meaningfully high levels but other areas are predicted to see no change, with the total area at risk in Europe increasing from 0.22% (0.08–0.51) in 2015 to just 0.62% (0.12–3.13) in 2080 (Fig. 3a), with any expansions in population at risk highly uncertain (Fig. 3b).

Globally we predict 2.25 (1.27–2.80) billion more people will be at risk of dengue in 2080 compared to 2015, bringing the total population at risk to over 6.1 (4.7–6.9) billion (Table 2), or 60% of the world’s population, according to RCP6.0/SSP2. This growth will be largely driven by population growth in already endemic areas as opposed to the spread of DENV to new populations, emphasizing the increasing public health burden many dengue endemic countries are likely to face. The future trajectory of global dengue depends highly on which RCP/SSP scenario is realised, with RCP4.5/SSP1 even suggesting decreases between 2050 and 2080 (Fig. 3c). This suggests that curbs in emissions and more sustainable socioeconomic growth targets offer hope of limiting the future impact of dengue.

Any long-term future projection is subject to a range of assumptions and limitations. In particular, we assume the stationarity of the effects and interactions of drivers of dengue transmission, and the absence of innovations and improvements in dengue control. There is also considerable uncertainty in the structure of the models used to predict future climate, *Aedes* distribution and dengue risk, which we have endeavoured to appropriately quantify and propagate by using ensembles of 17 different climate models and 100 dengue risk models. Despite these limitations, projections using the best possible compendium of dengue occurrence currently available have considerable public health value because detailed, systematically derived projections provide an evidence base that can be updated through time for prioritizing resources and informing long-term planning. Specifically, maps of environmental suitability are useful in helping identify where and when transmission can occur and has occurred^19^, and when and where seasonal peaks occur^20^.

Maps and projections may also be used for prioritizing vaccine dissemination, should this become a widely available option. Future work should include intervention scenarios based on current (insecticide and source reduction) and emerging (vaccine, sterile insect technique and *Wolbachia*) tools. A rapid global expansion of dengue poses a challenge to public health officials and policymakers alike, which we have shown is likely to occur under all climate scenarios; their actions today will have a significant impact on the future distribution of dengue. We have provided a set of maps (with appropriate caveats) identifying three possible futures for the global distribution of dengue until the year 2080. We have incorporated information about the spread of *Aedes* vectors, urbanization and population growth, showing that while climate change will be important, so will other drivers of disease distribution and abundance. Although there will be increases and decreases, we expect that the populations at greatest risk of this disease will grow substantially and disproportionately in the most economically disadvantaged areas.

## Methods

To create the 2015 map of environmental suitability for dengue occurrence, we applied an ecological niche modelling approach to establish a multivariate empirical relationship between the probability of dengue presence and the environmental conditions sampled at disease occurrence locations. Specifically, we used an ensemble boosted regression tree (BRT) methodology, which requires the generation of: (1) a comprehensive compendium of the locations of disease reports in humans; (2) a set of background points representing environmental conditions across the area of interest; and (3) a set of global gridded layers of environmental and socioeconomic covariates known or hypothesized to affect dengue transmission. The resulting model produces a 5 × 5-km spatial resolution global map of environmental suitability for dengue presence. By replacing the 2015 covariates with their respective 2020, 2050 or 2080 projections for three different future scenarios, a set of nine final map projections was made. The ensemble BRT approach and each of its components are described in greater detail below, with a description of our choice of future scenarios and how environmental and socioeconomic covariates were projected accordingly.

### Ensemble BRT approach

The BRT modelling procedure combines regression trees^21^ with gradient boosting^22^. In this procedure, an initial regression tree is fitted and iteratively improved upon in a forward stepwise manner (boosting) by minimizing the variation in the response variable not explained by the model at each iteration. This has been shown to fit complex non-linear response functions efficiently, while guarding against over-fitting. As such, this approach has been successfully used in the past to map dengue and its *Aedes* mosquito vectors, as well as many other infectious diseases^3,5,23–25^. To increase the robustness of model predictions and quantify model uncertainty, we fitted an ensemble^26^ of 100 BRT models to separate bootstraps of the data. We then evaluated the central tendency as the mean across all 100 BRT models^3^. Each of the 100 individual models was fitted using the ‘gbm.step’ subroutine to determine the number of trees that maximize the cross-validation predictive accuracy, as implemented in the ‘dismo’ package in the R statistical programming environment^27^. All other tuning parameters of the algorithm were held at their default values (tree complexity = 4, learning rate = 0.005, bag fraction = 0.75, step size = 10, cross-validation folds = 10). Each of the 100 models predict environmental suitability on a continuous scale from 0 to 1, with a final prediction map generated by calculating the mean prediction across all models for each 5 × 5-km pixel. Cross-validation was applied to each model bootstrap, whereby ten subsets of the data comprising 10% of the presence and background observations were assessed based on their ability to predict the distribution of the other 90% of records using the mean area under the receiver operating characteristic curve (AUC) statistic. The AUC value was averaged across the ten sub-models and finally across all 100 models in the ensemble to derive an overall estimate of goodness-of-fit. Additionally, to avoid AUC inflation due to spatial sorting bias, a pairwise distance sampling procedure was used^28^, resulting in a final AUC thatis lower than would be returned by standard procedures but that gives a more realistic quantification of the model’s ability to extrapolate predictions to new regions^29^.

### Assembly of the geo-referenced dengue occurrence database

The dengue occurrence database was first created as described previously^2^, with published literature, case report and informal online searches last updated on 27 July 2015. Usable location information was ultimately extracted from 2,229 published references and 1,648 informal online sources. This resulted in 35,467 entries that then underwent temporal standardization as described in ref. ^2^. The final occurrence database contained 13,604 unique occurrences, which represent a unique location where one or more dengue cases occurred within one year. As such, occurrences should not be interpreted as case counts, but individual unique locations may have experienced multiple occurrences over the years 1960–2015. A version of this database was initially published^2^ for records dating until early 2012, and the updated database containing information until the year 2015 can now be accessed via the same citation. A map of the final set of occurrence locations used for modelling the contemporary distribution of risk for dengue is provided in Supplementary Fig. 2a, with the number of occurrences per year globally and by region shown in Supplementary Fig. 2b.

### Generation of the background location dataset

To predict the distribution of dengue from occurrence records, we also need to provide the model with background records, representing environmental conditions across the area of interest. To reduce spatial bias in reporting of dengue occurrences, we followed previous work^30^ in using a target-group background approach to selecting these points. This method selects background points that are subject to similar spatial bias in reporting rates, so that the difference in distribution between occurrence and background records better reflects the true distribution of the disease; analogous to an epidemiological case-control analysis. These background points are, therefore, selected from the occurrence records of other diseases that are likely to be subject to similar spatial bias in reporting rates. As background points we used disease occurrence records from the HealthMap database for viral diseases that are principally diagnosed by serology and PCR, that cause general febrile illness, and for which at least 100 records were available. The spatial distribution of these disease reports therefore reflects both general spatial bias in disease reporting, as well as spatial variation in diagnostic capacity, for well-known diseases. This resulted in a total of 7,219 background records dated between 1960 and 2015, representing occurrence reports for Rift Valley fever, Japanese encephalitis, West Nile fever, yellow fever, Zika, Nipah, eastern equine encephalitis, viral meningitis and Crimean-Congo haemorrhagic fever.

### Explanatory covariates

Dengue virus transmission is maintained in urban settings where humans and mosquitoes are the only known hosts, with a sylvatic cycle occurring in non-human primates in forested areas and rarely resulting in transmission to humans^31–34^. Central to the global emergence of dengue virus has been the spread of its *Aedes* species mosquito vectors, in particular *Ae. aegypti* and, to a lesser degree, *Ae. albopictus*, which are both found across tropical and subtropical latitudes, as well as some parts of Europe and North America^5,35^. A complex interaction of factors influences the spatial distribution of these vectors, as well as their ability to transmit dengue. Environmental factors such as precipitation, humidity and temperature^36–39^ were most often incorporated into past efforts to model the distribution of dengue transmission. Much attention has also been given to the importance of socioeconomic factors in dengue transmission dynamics, including urban poverty, overcrowding, erratic water supply and poor public health infrastructure^6,40,41^, and these factors have more recently been incorporated into global distribution modelling of dengue^3,10^.

We included seven covariates in our species distribution modelling procedure. These covariates were chosen to reflect the factors known or hypothesized to be ecologically relevant to dengue virus transmission dynamics on a global scale, and for which it was feasible to collect data or derive proximate measures at a global scale for the year 2015 and project these measures to the years 2020, 2050 and 2080. The resulting set of covariates included the following: (1) annual cumulative precipitation; (2) minimum relative humidity; (3) an index of temperature suitability for DENV transmission; (4) the global distribution of *Ae. aegypti* mosquitoes; (5) the global distribution of *Ae. albopictus* mosquitoes; (6) GDP per 5 × 5-km cell; and (7) 5 × 5-km cells classified as urban or non-urban. Covariates 1–6 were also obtained or produced at a 5 × 5-km spatial resolution. All grids were standardized to ensure consistency of land classification. Maps for each covariate across all years and scenarios RCP6.0/SSP2 are provided in Supplementary Fig. 4, with raster datasets for covariates representing RCP4.5/SSP1 and RCP8.5/SSP3 available upon request.

### Climate scenarios and variables

In its Fifth Assessment Report (AR5), the IPCC used four representative concentration pathways (RCPs), each of which is characterized by fixed values for prescribed greenhouse gas concentrations by the year 2100^42,43^. The process in AR5 differs from previous assessments, which started with detailed socioeconomic storylines to generate emissions scenarios. In AR5, following a literature review, the IPCC combined different sets of potential economic, technological, demographic, policy and institutional futures, leading to the distinction of four RCPs (RCP8.5, RCP6.0, RCP4.5 and RCP2.6) representing the specific cumulative measure of radiative forcing (greenhouse gas emissions from all human sources) by the year 2100, expressed in watts per square metre (W m^−2^). The four distinguishable futures are intended as a set of potential narratives rather than forecasts or policy recommendations. RCP8.5 is characterized by increasing greenhouse gas emissions, representative of scenarios that lead to high greenhouse gas concentration levels^44^. RCP6.0 is considered a ‘stabilization scenario’ in which emissions peak around 2080 and then decline due to the application of a range of technologies and mitigating strategies, and stabilize shortly after 2100^45^. RCP4.5 is also a stabilization scenario, resulting from aggressive greenhouse gas reduction strategies and an emissions peak around 2040, with stabilization shortly after 2100^46,47^. RCP2.6 represents an aggressive mitigation scenario, with radiative forcing peaking at around 3.1 W m^−2^ by mid-century, and returning to 2.6 W m^−2^ by 2100^48^.

Many different global climate models (GCMs) of varying complexity have been used by the AR5. Each model predicts the climatic variables for a series of years, and for each of the RCP conditions^49^. In order to achieve the stabilization temperature target of below 2 °C by the year 2100, most climate models would require that global emissions of carbon dioxide peak around now and decline hereafter. Several models even require negative emissions by the second half of this century in order for this scenario to be achievable^50^. As such, in the present study we chose to present possible dengue futures based on RCPs 4.5, 6.0 and 8.5 only.

#### The WorldClim database

WorldClim v1.03 (http://www.wordclim.org) is derived from historic rainfall and temperature data (spanning the period 1961–2005, inclusive) collected from worldwide weather stations^51^. Data from these weather stations were then interpolated to 30 arc seconds using a thin plate smoothing algorithm in the ANUSPLIN-SPLINA software^52^. For the purpose of this study, these data were aggregated to 2.5 minute pixels as a standardized baseline for the GCM models.

#### GCM models

Monthly data from 17 GCM models (Supplementary Table 1) for the years 2000 to 2095 were converted to deviations from the relevant GCM baseline and interpolated from their native pixel size to a standard pixel size of 1° of latitude and longitude. The GCM data are all treated as differences from the individual model baselines and bias-corrected to current WorldClim values. All RCPs were represented in all GCMs. For each 1° pixel, the fifth-order polynomial regression was fitted over the 96 years and through the nominal origin of 1985 to calculate one output per model per year per scenario.

#### Ensemble calculation

Values are generated for each month for minimum and maximum temperature and for precipitation, taken independently from each GCM. We calculated the average regression coefficients for each of the 17 GCMs and downscaled to 2.5 minute pixels using a bi-cubic convolution algorithm from the MarkSim^53^ system as implemented in http://gisweb.ciat.cgiar.org/MarkSimGCM/docs/doc.html.

Predictions were made for each downscaled GCM for the three RCP regimes for the time points 2015, 2020, 2050 and 2080 using the common WorldClim baseline. From these monthly values, a series of annual mean summary indices that were GCM-, RCP- and year-specific were produced.

#### Annual cumulative precipitation

Presence of static surface water in natural or man-made containers is a prerequisite for *Aedes* oviposition and larval and pupal development. While fine-scale spatial and temporal heterogeneities have been observed between precipitation, vector abundance and dengue incidence, there is evidence that areas with greater amounts of precipitation are generally associated with higher dengue infection risk^54–60^. The annual cumulative precipitation (mm) was extracted for each 5 × 5-km grid cell globally for each GCM, RCP and year combination.

#### Temperature suitability for dengue transmission

Temperature affects key physiological processes including age and temperature-dependent adult female mosquito survival, as well as the duration of the extrinsic incubation period (EIP) of DENV and the length of gonotrophic cycle^61^. These parameters can and have been measured experimentally, allowing for the quantified effects of temperature on these mechanisms to be incorporated into a cohort simulation model^11^. This model analysed the cumulative effects of both diurnal and inter-seasonal changes in temperature on dengue transmission within an average year. The model was then applied to the monthly minimum and maximum temperature predictions for each CGM, RCP and year combination at a 5 × 5-km grid cell resolution, resulting in maps of temperature suitability for DENV transmission that range from 0 (no transmission possible) to 1 (most suitable pixel in the world). Areas with temperature suitability values of less than 14/365 = 0.04 were used to mask (exclude) future dengue risk predictions as they were unlikely to support transmission over the two week serial interval of autochthonous dengue transmission.

#### Minimum relative humidity

Greater relative humidity has been found to promote dengue virus growth in *Ae. aegypti* mosquitoes in several localized settings^62,63^, and has also been found to be an important contributor when predicting risk at a global scale^36^. We included the minimum annual relative humidity in our models as a potential limiting factor to dengue virus transmission. Relative humidity (RH) was calculated as a percentage of saturation humidity, or the amount of water vapour required to saturate the air given a particular temperature. The saturation, or ‘dew’ point (T_dew_), was calculated using the tabular relationship from ref. ^64,65^ RH was then calculated as follows: $RH = \frac{{V\left( {T_x} \right)}}{{V\left( {T_{dew}} \right)}} \times 100$

Where $V\left( {T_{dew}} \right) = 6.1121 \times {\mathrm{exp}}\left( {17.502 \times \frac{T}{{240.97 + T}}} \right)$ (from ref. ^63^) and $V_{T_x}$ is the humidity at the given temperature. We then extracted the minimum annual RH for each 5 × 5-km pixel globally for each GCM, RCP and year combination.

### Global maps of *Ae. aegypti* and *Ae. albopictus* presence

#### Environmental suitability

For the purpose of this study we built on a previously established modelling approach to produce global environmental suitability maps for *Ae. aegypti* and *Ae. albopictus*, the dominant DENV mosquito vectors^5^. One hundred BRT predictions of the global distribution of each species per GCM, RCP, and year (17 × 3 × 3 × 100 = 15,300 maps per species) were included in the covariate ensemble for the dengue future modelling, thus fully propagating uncertainty in the future distribution of *Aedes* species within our dengue model.

#### Current mosquito range

Environmental suitability is necessary, but not sufficient, for presence of a mosquito population. For a novel environmental suitability niche to be fully realized, mosquitoes need to be introduced into these novel areas through spread from their established range. The current (2015) global range for *Ae. aegypti* and *Ae. albopictus* was defined by taking the environmental suitability value that maximized sensitivity and specificity of classification of the occurrence and background data; this was found to equal 0.47 for *Ae. aegypti* and 0.51 for *Ae. albopictus*. Any value above this threshold was assumed to be within the range of the mosquito species.

#### Future mosquito range

To predict the future range of each mosquito species, connectivity matrices were calculated^12^ between all pixels within the mosquitoes’ range and all pixels outside the mosquitoes’ range, but with environmental suitability greater than 0. These fitted models were used to stochastically simulate mosquito spread each year (2015–2080) using the different baselines (calculations described previously) to give 100 potential future distributions for each future year. Areas where the mosquito was predicted to have less than 0.1% probability of presence (suitability × invasion probability) were excluded from the final dengue risk maps.

### Socioeconomic and population variables

Shared socioeconomic pathways (SSPs) were defined previously^66^ as reference pathways that describe plausible alternate trends in the evolution of society and ecosystems over a century timescale in the absence of climate change or climate policies. These SSPs are predicated on possible outcomes that would make it more or less difficult to respond to climate change challenges; as such, these can be combined with RCPs in a two-dimensional matrix to develop multiple scenarios of global change to the year 2100^67^. It is only by combining the two types of pathways that comprehensible depictions of the future can be constructed. Previous work^68^ suggested a set of suitable combinations of RCPs and SSPs that would allow for approximate comparison to the former scenarios presented in the 2000 IPCC Special Report on Emissions Scenarios (SRES)^69^. Based on these recommendations, we chose the following combinations for our projections: (1) RCP8.5 with SSP3 to correspond with the SRES A2 world (increasing population and regionally-oriented economic development); (2) RCP6.0 with SSP2 to correspond with the SRES A1B/B2 world (both characterized by low population growth and technological change); and (3) RCP4.5 with SSP1 to correspond with the SRES B1 world (global economic convergence and the introduction of resource-efficient technologies). Each SSP consists of quantified population and Gross Domestic Product (GDP) trajectories, serving as the starting point for various organisations to model these factors and provide projections for demographic and economic development variables. The Integrated Assessment Modelling Consortium (IAMC) made available certain peer-reviewed projections via the International Institute for Applied Systems Analysis (IIASA, http://www.iiasa.ac.at), whereby the SSP storylines were converted into population and GDP projections for 195 countries^70–72^ for every decade between the years 2010 and 2100. Using these projections, we carried out the procedures described next in order to incorporate GDP per 5 × 5-km cell in our dengue models and projections.

#### GDP per cell

We started with a 2010 population density raster at a 5 × 5-km resolution that was a hybrid of WorldPop population density estimates (http://www.worldpop.org.uk) where available, and version 4 of the Global Rural-Urban Mapping Project (GRUMP; http://sedac.ciesin.columbia.edu) population density maps where not available. For each of SSPs 1–3, the following steps were taken: (1) for countries with population projections, we calculated the national population growth rates from the IIASA database between each pair of years in 2010, 2015, 2020, 2050 and 2080; (2) for countries with no population projections available, we calculated the average population growth rate over the period from other countries in the same region as described earlier for GDP; (3) we produced 5 × 5-km rasters of the national-level populations for 2015, 2020, 2050 and 2080 by multiplying all cells in each country by the national-level growth rates for each scenario year combination; (4) for all countries with projections in the IIASA dataset, we next calculated GDP per capita by dividing GDP by the total population for each scenario (SSPs 1–3) and year (2015, 2020, 2050 and 2080), averaging across scenarios for the year 2015; and (5) finally, for each scenario and year, we in-filled GDP per capita for countries with missing data using a regional mean according to the IIASA-defined regions (North America, Latin America, Western Europe, Central Asia, Eastern Europe, Middle East, European FSU and North Africa). We were then able to link the data for all countries to a raster map of country identifiers for all scenario year combinations in order to multiply GDP per capita by population density and produce GDP per 5 × 5-km cell.

#### Urban extents

The spatial distribution of predicted global urban expansion was modelled according to previous work undertaken for Africa^73^. In addition, the same methodologies were recently used to map urban land expansion between 2000 and 2010 in Asia^74^. The methods were modified for ease of replication to any country and the spatial resolution of the input and output datasets was increased to 5 × 5-km. In brief, each pixel was characterized for its likelihood of becoming urban in an ensemble BRT framework using the year 2000 urban extents from the Moderate Resolution Imaging Spectroradiometer Collection 5 (MODIS C5) land-cover product^75,76^ and several covariates hypothesized to be associated with urban habitat type. Covariates included: (1) the travel time to the closest pixel classified as urban^77^; (2) the proportion of urbanized land within 20 km; (3) human population density as described earlier; (4) slope, derived from the US Geological Survey SRTM30 dataset^78^; and (5) the distance to water, calculated using a land/sea mask and inland water layer from the National Geospatial-Intelligence Agency (NGA) Vector Map Level 0 (VMAP0) data. BRT models were developed to predict the rural-urban conversion probability for each 5 ×-5 km pixel using the covariates described above. Urban extents were first simulated from 2000 (T0) to 2010 (T1), and predicted urban extents in T1 were then used as a baseline to predict urban change for the next decade iteratively up to 2080. The rural-urban conversion probability raster was then used to probabilistically allocate new urban development, while preventing new urban development from occurring in water bodies and protected areas^79^.

#### Dengue future modelling ensemble approach

Our final aim was to produce nine maps, a prediction for dengue suitability in the years 2020, 2050 and 2080 under three different emissions scenarios (RCPs). Each of these nine maps were composed of 100 ensemble predictions that randomly sampled (with replacement) the following aspects of the analysis:

1. The fitted dengue BRT model (from a choice of 100 BRT models fitted to 2015 data).
2. The predicted future distribution of *Ae. aegypti* (from a choice of 100 model predictions^12^).
3. The predicted future distribution of *Ae. albopictus* (from a choice of 100 model predictions^12^).
4. The predicted temperature suitability for dengue transmission (from a choice of 17 GCMs).
5. The predicted minimum monthly precipitation (from a choice of 17 GCMs).
6. The predicted relative humidity (from a choice of 17 GCMs).
7. The predicted maximum monthly precipitation (from a choice of 17 GCMs).

This approach sought to fully propagate the uncertainty in the climate, *Aedes* and dengue models through to the final prediction (see maps of uncertainty estimates in Supplementary Fig. 5). These 100 predictions were then summarized by mean and 95% credible intervals to give the final prediction for each year RCP combination.

#### Calculating population and area at risk of dengue transmission

To classify areas as at risk or not at risk using the continuous dengue suitability maps, a threshold was defined by the value that maximized sensitivity and specificity when classifying the occurrence and background data using the 2015 map. Any pixel with a predicted dengue suitability value above 0.467 was considered at risk and the same threshold was applied to each time point and scenario to calculate the population and area at risk in each global region. This procedure was carried out independently for each of the 100 predictions arising from each of the RCPs and timepoints to propagate uncertainty into these global statistics.

### Reporting Summary

Further information on research design is available in the Nature Research Reporting Summary linked to this article.

## Supplementary information

Supplementary Information Supplementary Figures 1–6, Supplementary Table 1 and Supplementary References.

Reporting Summary

## Data Availability

Data are available from: https://figshare.com/s/d7d7871d00afe2870619
