# Supplementary material for: The current and future global distribution and population at risk of dengue
Source: Nat Microbiol. 2019 Jun 10;4(9):1508–15. doi: 10.1038/s41564-019-0476-8 (PMC6784886; doi:10.1038/s41564-019-0476-8)
Supplement: Supplementary file 1 — Supplementary Figures 1–6, Supplementary Table 1 and Supplementary References. [file 41564_2019_476_MOESM1_ESM.pdf]

nature microbiology

SUPPLEMENTARY INFORMATION
https://doi.org/10.1038/s41564-019-0476-8

In the format provided by the authors and unedited.

The current and future global distribution and population at risk of dengue

Jane P. Messina[1,2,17*], Oliver J. Brady[3,4,17], Nick Golding[5], Moritz U. G. Kraemer[6,7,8], G. R. William Wint[9], Sarah E. Ray[10], David M. Pigott[10], Freya M. Shearer[11], Kimberly Johnson[10], Lucas Earl[10], Laurie B. Marczak[10], Shreya Shirude[10], Nicole Davis Weaver[10], Marius Gilbert[12], Raman Velayudhan[13], Peter Jones[14], Thomas Jaenisch[15], Thomas W. Scott[16], Robert C. Reiner Jr[10,18] and Simon I. Hay[10,18*]

[1]School of Geography and the Environment, University of Oxford, Oxford, UK. [2]School of Interdisciplinary Area Studies, University of Oxford, Oxford, UK. [3]Centre for the Mathematical Modelling of Infectious Diseases, London School of Hygiene & Tropical Medicine, London, UK. [4]Department of Infectious Disease Epidemiology, London School of Hygiene & Tropical Medicine, London, UK. [5]School of BioSciences, University of Melbourne, Parkville, VIC, Australia. [6]Harvard Medical School, Harvard University, Boston, MA, USA. [7]Boston Children's Hospital, Boston, MA, USA. [8]Department of Zoology, University of Oxford, Oxford, UK. [9]Environmental Research Group Oxford, c/o Department of Zoology, University of Oxford, Oxford, UK. [10]Institute for Health Metrics and Evaluation, University of Washington, Seattle, WA, USA. [11]Big Data Institute, Li Ka Shing Centre for Health Information and Discovery, University of Oxford, Oxford, UK. [12]Université Libre de Bruxelles, Brussels, Belgium. [13]World Health Organization, Geneva, Switzerland. [14]Waen Associates Ltd, Y Waen, Islaw'r Dref, Dolgellau, Gwynedd, UK. [15]Department of Infectious Diseases, Section Clinical Tropical Medicine, Heidelberg University Hospital, Heidelberg, Germany. [16]Department of Entomology and Nematology, University of California, Davis, USA. [17]These authors contributed equally: Jane P. Messina and Oliver J. Brady. [18]These authors jointly supervised this work: Robert C. Reiner Jr and Simon I. Hay. *e-mail: jane.messina@ouce.ox.ac.uk; sihay@uw.edu

Contents

Supplementary Figure 1: Schematic of modelling procedure for mapping present and future environmental suitability for dengue. 3

Supplementary Figure 2: (a) locations of the 13,604 occurrence locations used in the Boosted Regression Tree modelling procedure; (b) number of unique dengue occurrence locations per year according to world region. ... 4

Supplementary Figure 3: Effect plots for covariates entered into the ensemble of Boosted Regression Tree models, including (a) environmental suitability for *Aedes aegypti* (ranging 0-1); (b) environmental suitability for *Ae. albopictus* (ranging 0-1); (c) Gross Domestic Product per 5km x 5km gridded cell (USD); (d) probability of urban habitat type (ranging 0-1); (e) minimum relative humidity (proportion saturation humidity ranging 0-1,000) (f) annual cumulative precipitation (mm); (g) temperature suitability for dengue transmission (ranging 0-1). Shaded areas indicate the 95% confidence interval around predictions for the ensemble of 100 models run. 5

Supplementary Figure 4: 5 x 5km covariate maps for layers entered into the ensemble of Boosted Regression Tree models for 2015. (a) environmental suitability for *Aedes aegypti*; (b) environmental suitability for *Ae. albopictus;* (c) Gross Domestic Product per cell in $USD millions; (d) annual cumulative precipitation in mm; (e) minimum relative humidity; (f) temperature suitability for dengue transmission; (g) urban habitat probability.... 6

Supplementary Figure 5: Maps of uncertainty in suitability estimates, shaded according to suitability estimates and uncertainty. Areas in white have low uncertainty and low suitability, while areas in purple have high uncertainty and high suitability. Pink areas have low uncertainty and high suitability, while blue areas have low suitability and high uncertainty. 7

Supplementary Figure 6: Predicted Temperature Suitability Index (TSI) at different fixed temperatures as determined by the model from Brady et al. The optimal temperatures for dengue transmission are predicted to be 34.0 °C and 30.6 °C for *Ae. aegypti* and *Ae. albopictus* respectively which fall in the middle-to-upper range within previous temperature- based dengue transmission models. 8

Supplementary Table 1: Global Climate Models used for projection of climate variables. 9

Supplementary References 10

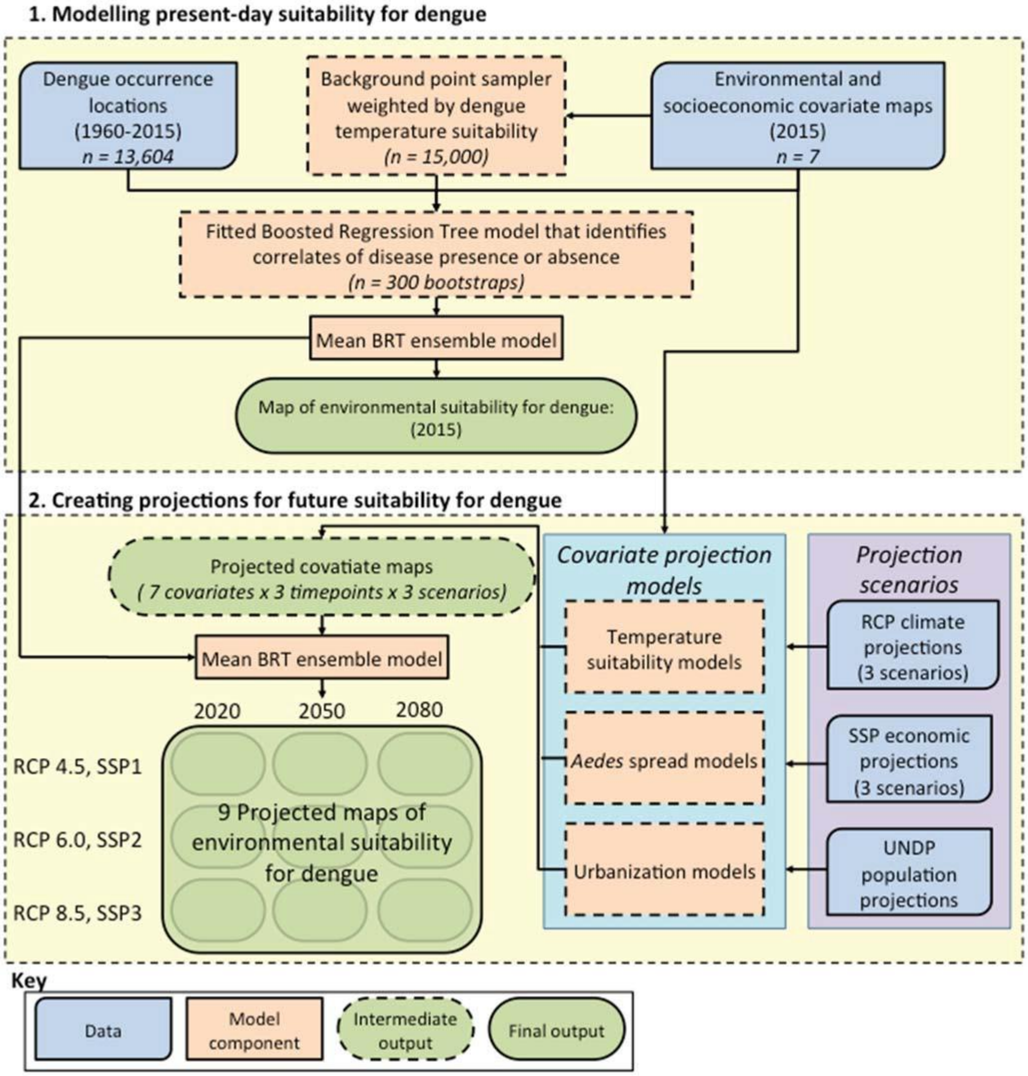

Supplementary Figure 1: Schematic of modelling procedure for mapping present and future environmental suitability for dengue.

A

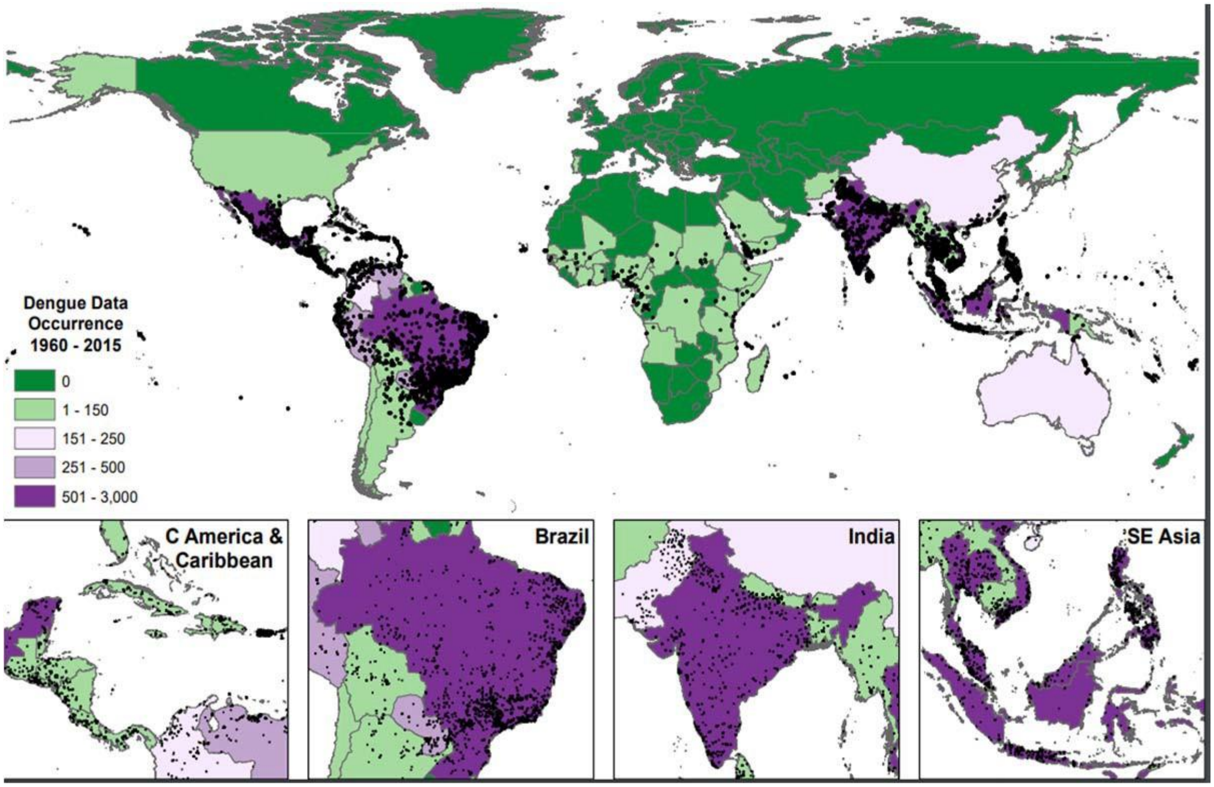

B

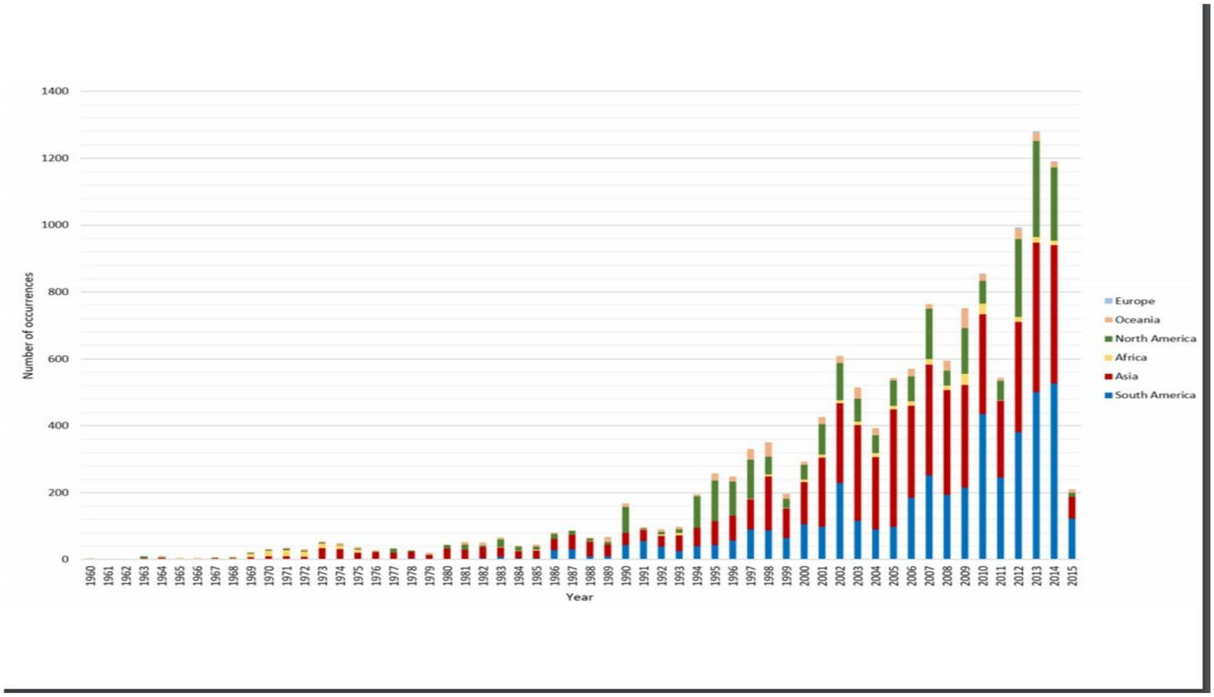

Supplementary Figure 2: (a) locations of the 13,604 occurrence locations used in the Boosted Regression Tree modelling procedure; (b) number of unique dengue occurrence locations per year according to world region.

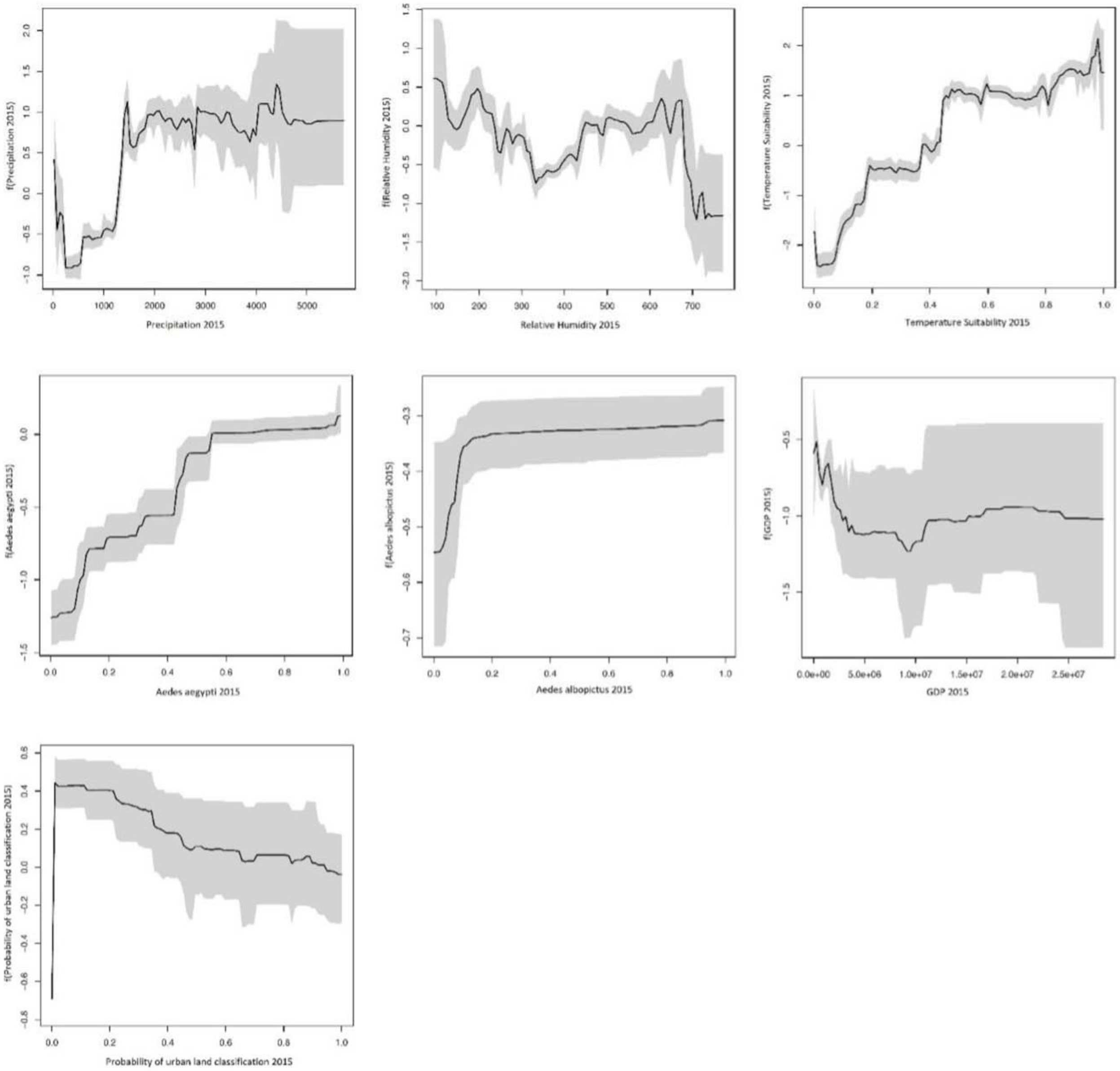

Supplementary Figure 3: Effect plots for covariates entered into the ensemble of Boosted Regression Tree models, including (a) environmental suitability for *Aedes aegypti* (ranging 0-1); (b) environmental suitability for *Ae. albopictus* (ranging 0-1); (c) Gross Domestic Product per 5km x 5km gridded cell (USD); (d) probability of urban habitat type (ranging 0-1); (e) minimum relative humidity (proportion saturation humidity ranging 0-1,000) (f) annual cumulative precipitation (mm); (g) temperature suitability for dengue transmission (ranging 0-1). Shaded areas indicate the 95% confidence interval around predictions for the ensemble of 100 models run.

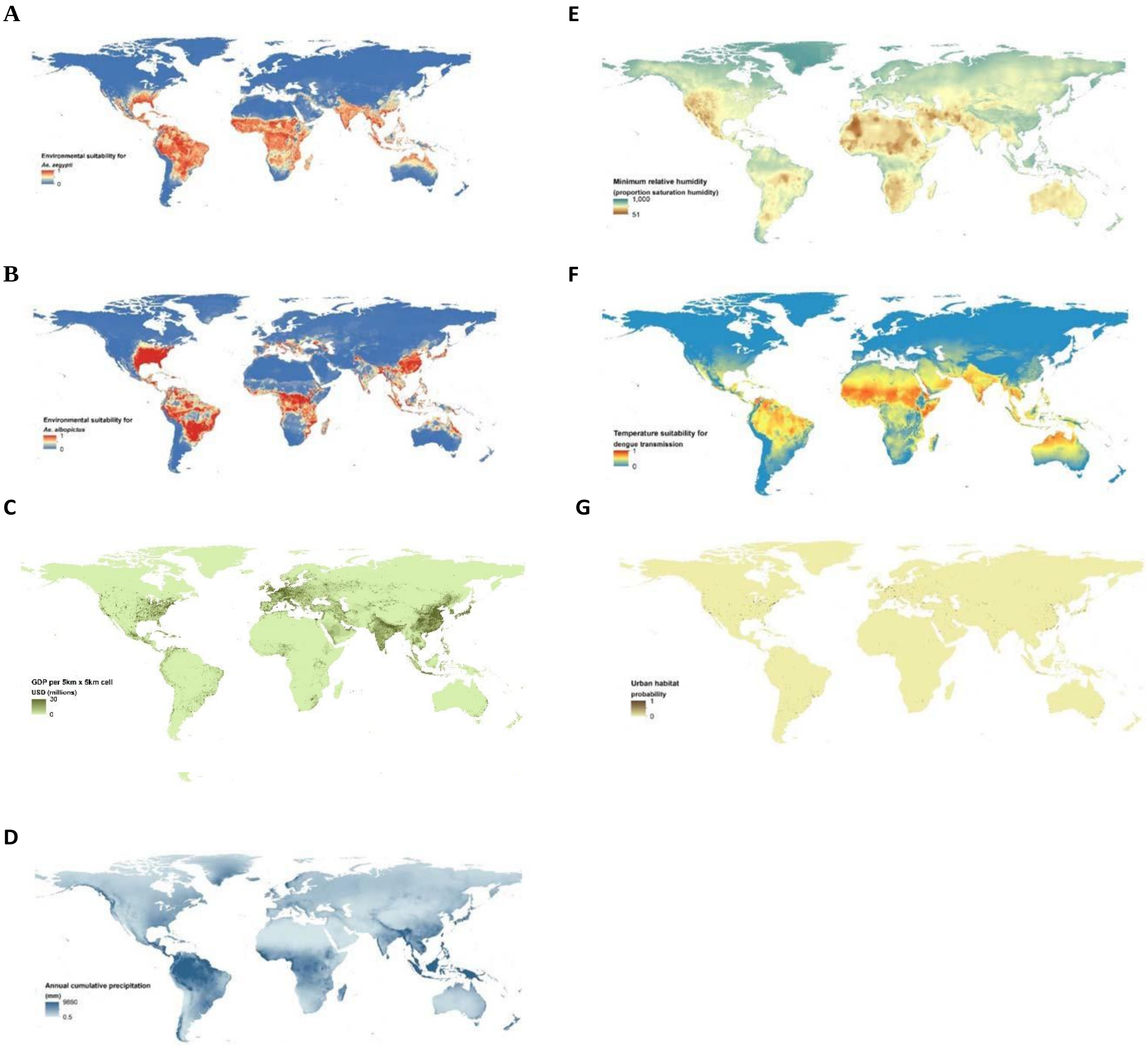

Supplementary Figure 4: 5 x 5km covariate maps for layers entered into the ensemble of Boosted Regression Tree models for 2015. (a) environmental suitability for *Aedes aegypti*; (b) environmental suitability for *Ae. albopictus;* (c) Gross Domestic Product per cell in $USD millions; (d) annual cumulative precipitation in mm; (e) minimum relative humidity; (f) temperature suitability for dengue transmission; (g) urban habitat probability.

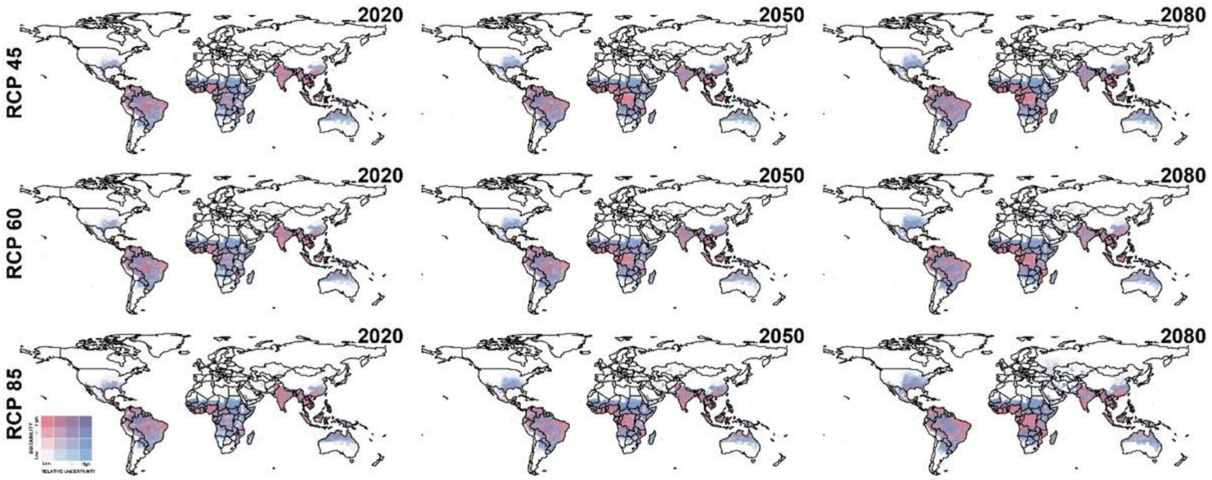

Supplementary Figure 5: Maps of uncertainty in suitability estimates, shaded according to suitability estimates and uncertainty. Areas in white have low uncertainty and low suitability, while areas in purple have high uncertainty and high suitability. Pink areas have low uncertainty and high suitability, while blue areas have low suitability and high uncertainty.

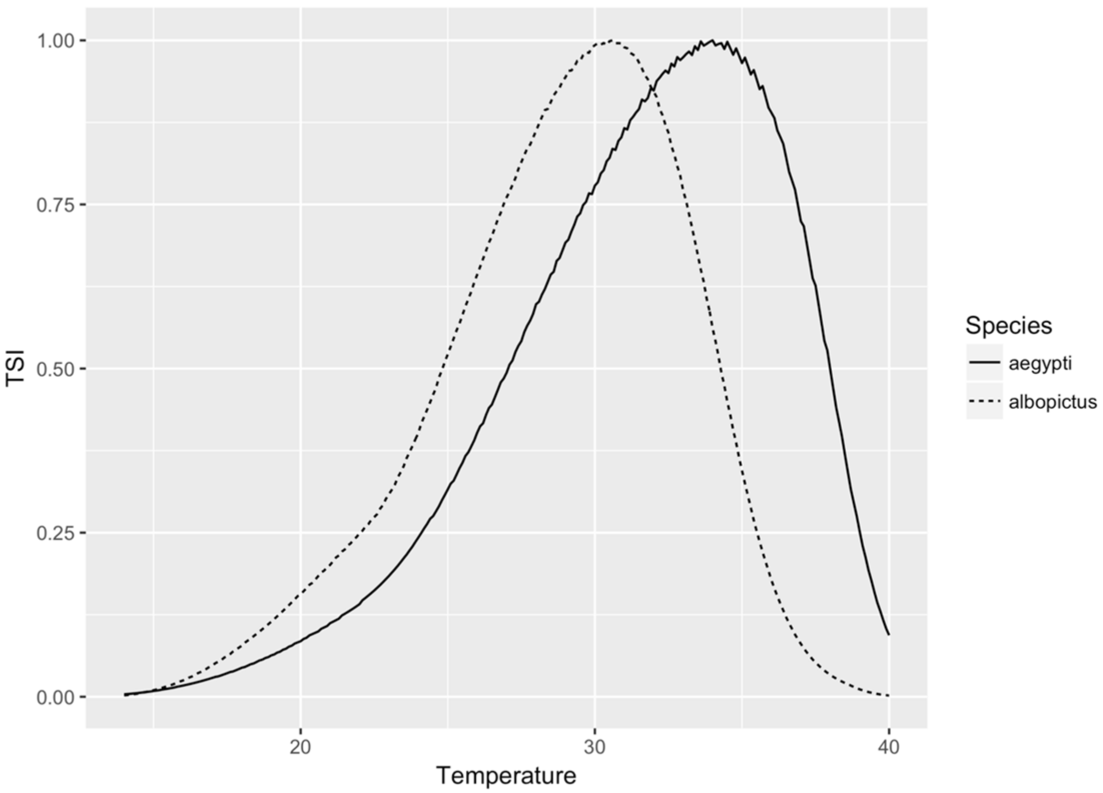

Supplementary Figure 6: Predicted Temperature Suitability Index (TSI) at different fixed temperatures as determined by the model from Brady et al. The optimal temperatures for dengue transmission are predicted to be 34.0˚C and 30.6˚C for *Ae. aegypti* and *Ae. albopictus* respectively which fall in the middle-to-upper range within previous temperature- based dengue transmission models.

Supplementary Table 1: Global Climate Models used for projection of climate variables.

	Model	Institution	Resolution, Lat x Long	Reference (see supplementary references for full citation)
1	BCC-CSM 1.1	Beijing Climate Center, China Meteorological Administration	2.8125 x 2.8125	Wu T (2012
2	BCC-CSM 1.1(m)	Beijing Climate Center, China Meteorological Administration	2.8125 x 2.8125	Wu T (2012
3	CSIRO-Mk3.6.0	Commonwealth Scientific and Industrial Research Organisation and the Queensland Climate Change Centre of Excellence	1.875 x 1.875	Collier MA et al. (2011)
4	FIO-ESM	The First Institute of Oceanography, SOA, China	2.812 x 2.812	Song Z, Qiao F, Song Y (2012)
5	GFDL-CM3	Geophysical Fluid Dynamics Laboratory	2.0 x 2.5	Donner LJ et al. (2011
6	GFDL-ESM2G	Geophysical Fluid Dynamics Laboratory	2.0 x 2.5	Dunne JP et al. (2012)
7	GFDL-ESM2M	Geophysical Fluid Dynamics Laboratory	2.0 x 2.5	Dunne JP et al. (2012)
8	GISS-E2-H	NASA Goddard Institute for Space Studies	2.0 x 2.5	Schmidt GA et al. (2006)
9	GISS-E2-R	NASA Goddard Institute for Space Studies	2.0 x 2.5	Schmidt GA et al. (2006)
10	HadGEM2-ES	Met Office Hadley Centre	1.2414 x 1.875	Collins WJ et al. (2011)
11	IPSL-CM5A-LR	Institut Pierre-Simon Laplace	1.875 x 3.75	Dufresne JL et al. (2013)
12	IPSL-CM5A-MR	Institut Pierre-Simon Laplace	1.2587 x 2.5	Dufresne JL et al. (2013)
13	MIROC-ESM	Atmosphere and Ocean Research Institute (The University of Tokyo), National Institute for Environmental Studies, and Japan Agency for Marine-Earth Science and Technology	2.8125 x 2.8125	Watanabe S et al. (2011)
14	MIROC-ESM-CHEM	Atmosphere and Ocean Research Institute (The University of Tokyo), National Institute for Environmental Studies, and Japan Agency for Marine-Earth Science and Technology	2.8125 x 2.8125	Watanabe S et al. (2011)
15	MIROC5	Japan Agency for Marine-Earth Science and Technology, Atmosphere and Ocean Research Institute (The University of Tokyo), and National Institute for Environmental Studies	1.4063 x 1.4063	Watanabe M et al. (2010)
16	MRI-CGCM3	Meteorological Research Institute	1.125 x 1.125	Yukimoto S (2012)
17	NorESM1-M	Norwegian Climate Centre	1.875 x 2.5	Kirkevag A, et al. (2008)<br>Seland O, et al. (2008)

Supplementary References

Collier MA et al. (2011) The CSIROMk3.6.0 Atmosphere-Ocean GCM: participation in CMIP5 and data publication. MODSIM 2011, Perth, 12–16 December 2011.

Collins WJ et al. (2011). Development and evaluation of an Earth-System model-HadGEM2. GMD 4(4):1051–1075.

Donner LJ et al. (2011). The dynamical core, physical parameterizations, and basic simulation characteristics of the atmospheric component AM3 of the GFDL Global Coupled Model CM3. Journal of Climate, 24(13).

Dufresne JL et al. (2013). Climate change projections using the IPSL-CM5 Earth System Model: from CMIP3 to CMIP5. Climate Dynamics, 1-43.

Dunne JP et al. (2012). GFDL's ESM2 Global Coupled Climate–Carbon Earth System Models. Part I: Physical Formulation and Baseline Simulation Characteristics. J. Climate, 25, 6646–6665.

Jones, P. G. and Thornton, P. K. (2013) Generating downscaled weather data from a suite of climate models for agricultural modelling applications. Agric. Systems. 114 (2013) 1-5.

Kirkevag A, Iversen T, Seland O, Debernard JB, Storelvmo T, Kristjansson JE (2008) Aerosol-cloud-climate interactions in the climate model CAM-Oslo.Tellus A 60(3):492–512.

Schmidt GA et al. (2006). Present day atmospheric simulations using GISS ModelE: Comparison to in-situ, satellite and reanalysis data. J. Climate 19, 153-192.

Seland O, Iversen T, Kirkevag A, Storelvmo T (2008). Aerosol-climate interactions in the CAM-Oslo atmospheric GCM and investigation of associated basic shortcomings. Tellus A 60(3):459–491.

Song Z, Qiao F, Song Y (2012). Response of the equatorial basin-wide SST to wave mixing in a climate model: An amendment to tropical bias, J. Geophys. Res., 117, C00J26.

Watanabe M et al. (2010). Improved Climate Simulation by MIROC5: Mean States, Variability, and Climate Sensitivity. J. Climate, 23, 6312–6335.

Watanabe S et al. (2011). MIROC-ESM2010: model description and basic results of CMIP5-20c3m experiments. Geoscientific Model Development 4 (4), 845–872.

Wu T (2012). A Mass-Flux Cumulus Parameterization Scheme for Largescale Models: Description and Test with Observations. Clim. Dynam. 38, 725–744.

Yukimoto S (2012). A new global climate model of Meteorological Research Institute: MRI-CGCM3 – Model description and basic performance. J. Meteorol. Soc. Jpn., 90a, 23–64.
