# Supplementary material for: The current and future global distribution and population at risk of dengue
Source: Nat Microbiol. 2019 Jun 10;4(9):1508–15. doi: 10.1038/s41564-019-0476-8 (PMC6784886; doi:10.1038/s41564-019-0476-8)
Supplement: Supplementary file 2 — Reporting Summary [file 41564_2019_476_MOESM2_ESM.pdf]

nature research

Corresponding author(s): J. Messina and S.I. Hay

Reporting Summary

Nature Research wishes to improve the reproducibility of the work that we publish. This form provides structure for consistency and transparency in reporting. For further information on Nature Research policies, see Authors & Referees and the Editorial Policy Checklist.

Statistical parameters

When statistical analyses are reported, confirm that the following items are present in the relevant location (e.g. figure legend, table legend, main text, or Methods section).

n/a	Confirmed	
☐	☒	The exact sample size (n) for each experimental group/condition, given as a discrete number and unit of measurement
☐	☒	An indication of whether measurements were taken from distinct samples or whether the same sample was measured repeatedly
☒	☐	The statistical test(s) used AND whether they are one- or two-sided<br>*Only common tests should be described solely by name; describe more complex techniques in the Methods section.*
☐	☒	A description of all covariates tested
☐	☒	A description of any assumptions or corrections, such as tests of normality and adjustment for multiple comparisons
☐	☒	A full description of the statistics including central tendency (e.g. means) or other basic estimates (e.g. regression coefficient) AND variation (e.g. standard deviation) or associated estimates of uncertainty (e.g. confidence intervals)
☒	☐	For null hypothesis testing, the test statistic (e.g. F, t, r) with confidence intervals, effect sizes, degrees of freedom and P value noted<br>*Give P values as exact values whenever suitable.*
☒	☐	For Bayesian analysis, information on the choice of priors and Markov chain Monte Carlo settings
☐	☒	For hierarchical and complex designs, identification of the appropriate level for tests and full reporting of outcomes
☒	☐	Estimates of effect sizes (e.g. Cohen's d, Pearson's r), indicating how they were calculated
☐	☒	Clearly defined error bars<br>*State explicitly what error bars represent (e.g. SD, SE, CI)*

Our web collection on statistics for biologists may be useful.

Software and code

Policy information about availability of computer code

Data collection	No primary data collection was carried out for this analysis.
Data analysis	WorldClim v1.03 (http://www.wordclim.org) is derived from historic rainfall and temperature data (spanning the period 1961-2005, inclusive) collected from worldwide weather stations. Data from these weather stations were then interpolated to 30 arc seconds using a thin plate smoothing algorithm in the ANUSPLIN-SPLINA software. We calculated the average regression coefficients for each of the 17 Global Climate Models and downscaled to 2.5 minute pixels using a bi-cubic convolution algorithm from the MarkSim50 system as implemented in http://gisweb.ciat.cgiar.org/MarkSimGCM/docs/doc.html. Data were then projected to three time point in the future 2020, 2050 and 2080. The models were all fit using R version 3.5.1.

For manuscripts utilizing custom algorithms or software that are central to the research but not yet described in published literature, software must be made available to editors/reviewers upon request. We strongly encourage code deposition in a community repository (e.g. GitHub). See the Nature Research guidelines for submitting code & software for further information.

Data

Policy information about availability of data

All manuscripts must include a data availability statement. This statement should provide the following information, where applicable:

- Accession codes, unique identifiers, or web links for publicly available datasets
- A list of figures that have associated raw data
- A description of any restrictions on data availability

Data used were previously archived as described in Kraemer, M. U. G. et al. The global compendium of Aedes aegypti and Ae . albopictus occurrence. Sci. Data 2, 150035 (2015). Other data were accessed from other published sources as described in the methods sections 1.1-1.4 on data availability. We accessed human mobility data from the freely available dataset at http://www.census.gov/hhes/commuting/.

Field-specific reporting

Please select the best fit for your research. If you are not sure, read the appropriate sections before making your selection.

☒ Life sciences ☐ Behavioural & social sciences ☐ Ecological, evolutionary & environmental sciences

For a reference copy of the document with all sections, see nature.com/authors/policies/ReportingSummary-flat.pdf

Life sciences study design

All studies must disclose on these points even when the disclosure is negative.

Sample size	The dengue occurrence database was first created as described in Messina et al (citation 2), with published literature, case report, and informal online searches last updated on 27th July, 2015. Usable location information was ultimately extracted from 2,229 published references and 1,648 informal online sources. This resulted in 35,467 entries which then underwent temporal standardization as described in detail in Messina et al. The final occurrence database contained 13,604 unique occurrences.
Data exclusions	After data cleaning described in Messina et al. no data were excluded.
Replication	This is an observational study using many years of survey data and could be replicated.
Randomization	As an observational mapping project, there were no experimental groups.
Blinding	Blinding was not relevant to this study, as it was an observational study using survey data.

Reporting for specific materials, systems and methods

Materials & experimental systems

n/a	Involved in the study
☒	☐ Unique biological materials
☒	☐ Antibodies
☒	☐ Eukaryotic cell lines
☒	☐ Palaeontology
☒	☐ Animals and other organisms
☒	☐ Human research participants

Methods

n/a	Involved in the study
☒	☐ ChIP-seq
☒	☐ Flow cytometry
☒	☐ MRI-based neuroimaging
